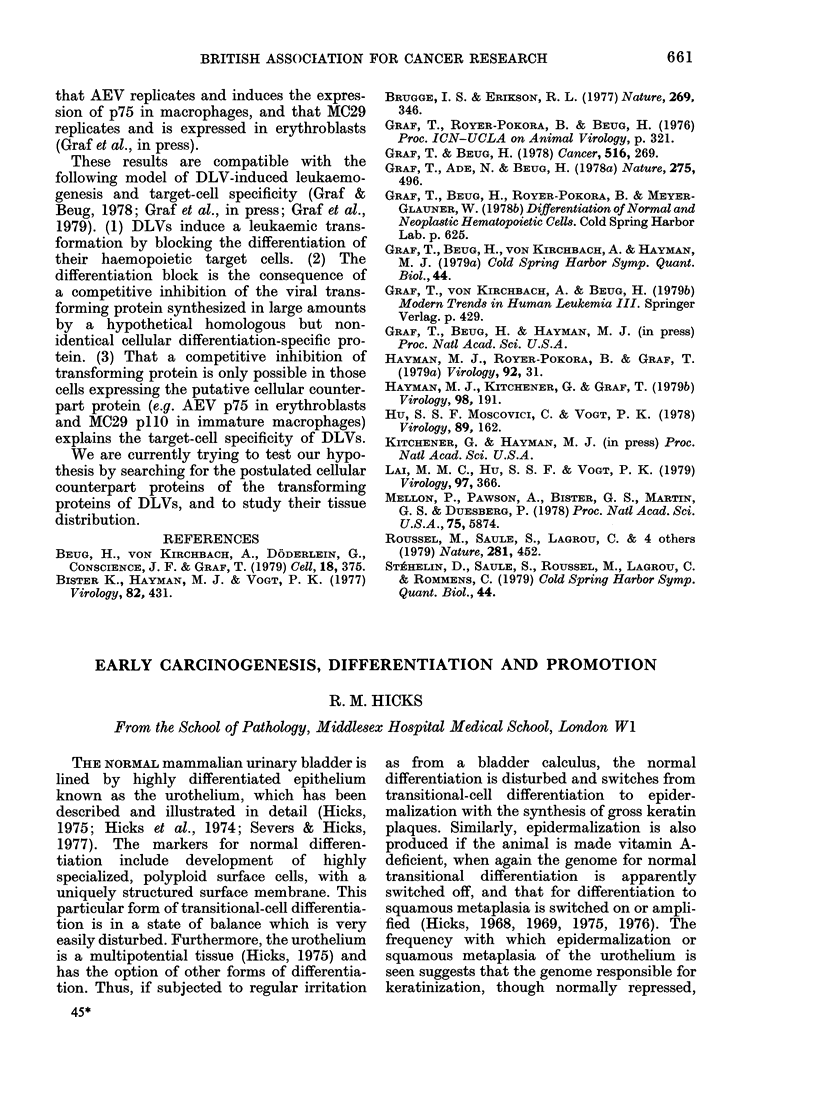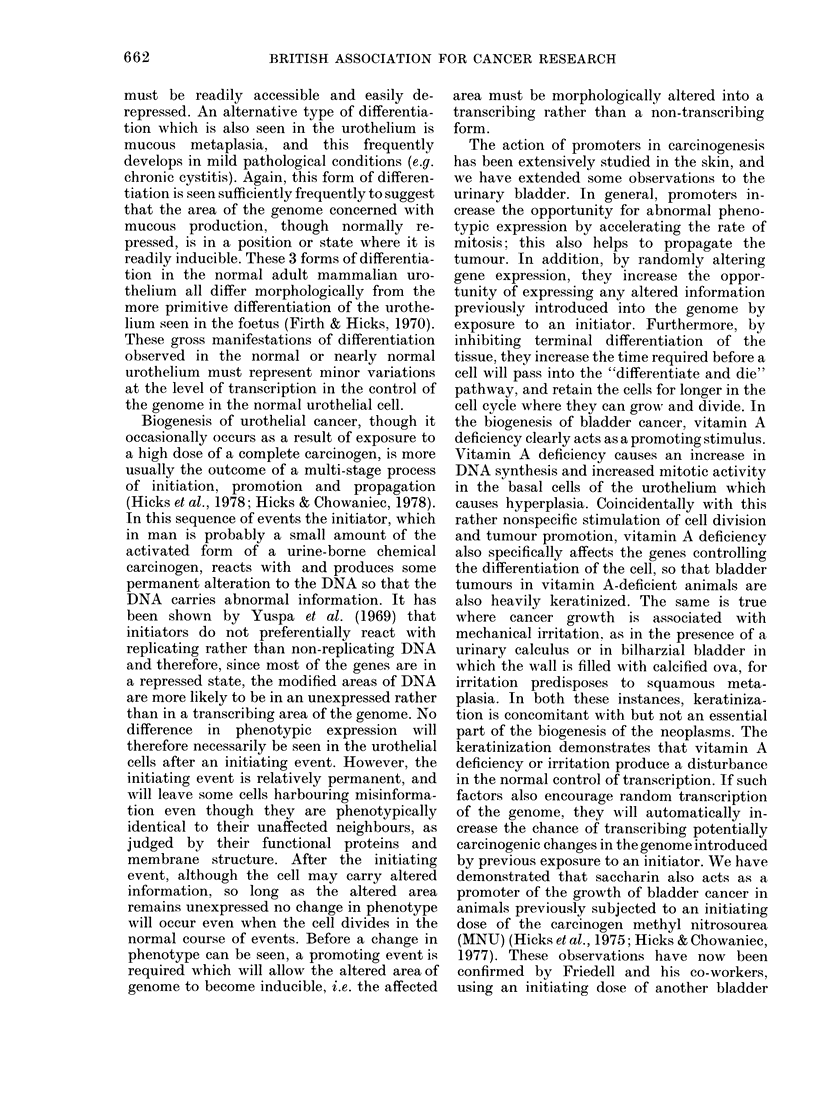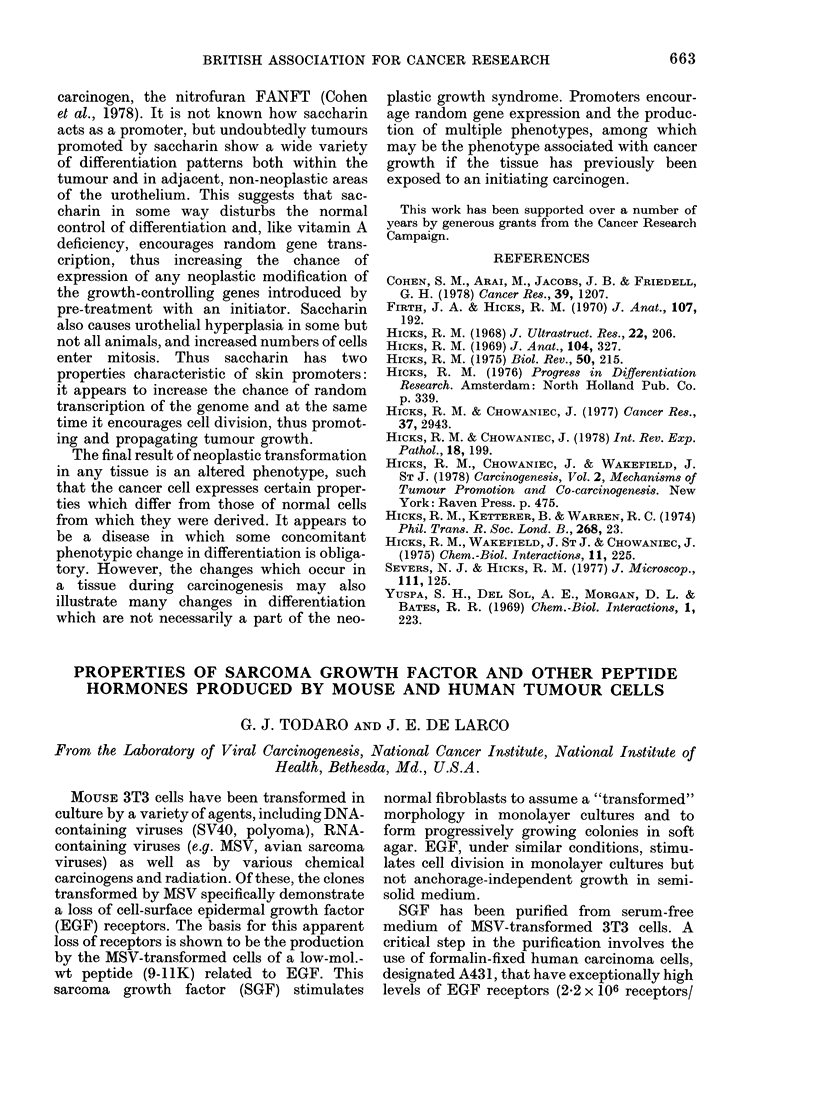# Early carcinogenesis, differentiation and promotion.

**DOI:** 10.1038/bjc.1980.115

**Published:** 1980-04

**Authors:** R. M. Hicks


### EARLY CARCINOGENESIS, DIFFERENTIATION AND PROMOTION

R. M. HICKS

*From the School of Pathology, Middlesex Hospital Medical School, London W1*

The normal mammalian urinary bladder is lined by highly differentiated epithelium known as the urothelium, which has been described and illustrated in detail (Hicks, 1975; Hicks *et al.*, 1974; Severs & Hicks, 1977). The markers for normal differentiation include development of highly specialized, polyploid surface cells, with a uniquely structured surface membrane. This particular form of transitional-cell differentiation is in a state of balance which is very easily disturbed. Furthermore, the urothelium is a multipotential tissue (Hicks, 1975) and has the option of other forms of differentiation. Thus, if subjected to regular irritation as from a bladder calculus, the normal differentiation is disturbed and switches from transitional-cell differentiation to epidermalization with the synthesis of gross keratin plaques. Similarly, epidermalization is also produced if the animal is made vitamin A-deficient, when again the genome for normal transitional differentiation is apparently switched off, and that for differentiation to squamous metaplasia is switched on or amplified (Hicks, 1968, 1969, 1975, 1976). The frequency with which epidermalization or squamous metaplasia of the urothelium is seen suggests that the genome responsible for keratinization, though normally repressed, must be readily accessible and easily derepressed. An alternative type of differentiation which is also seen in the urothelium is mucous metaplasia, and this frequently develops in mild pathological conditions (*e.g.* chronic cystitis). Again, this form of differentiation is seen sufficiently frequently to suggest that the area of the genome concerned with mucous production, though normally repressed, is in a position or state where it is readily inducible. These 3 forms of differentiation in the normal adult mammalian urothelium all differ morphologically from the more primitive differentiation of the urothelium seen in the foetus (Firth & Hicks, 1970). These gross manifestations of differentiation observed in the normal or nearly normal urothelium must represent minor variations at the level of transcription in the control of the genome in the normal urothelial cell.

Biogenesis of urothelial cancer, though it occasionally occurs as a result of exposure to a high dose of a complete carcinogen, is more usually the outcome of a multi-stage process of initiation, promotion and propagation (Hicks *et al.*, 1978; Hicks & Chowaniec, 1978). In this sequence of events the initiator, which in man is probably a small amount of the activated form of a urine-borne chemical carcinogen, reacts with and produces some permanent alteration to the DNA so that the DNA carries abnormal information. It has been shown by Yuspa *et al.* (1969) that initiators do not preferentially react with replicating rather than non-replicating DNA and therefore, since most of the genes are in a repressed state, the modified areas of DNA are more likely to be in an unexpressed rather than in a transcribing area of the genome. No difference in phenotypic expression will therefore necessarily be seen in the urothelial cells after an initiating event. However, the initiating event is relatively permanent, and will leave some cells harbouring misinformation even though they are phenotypically identical to their unaffected neighbours, as judged by their functional proteins and membrane structure. After the initiating event, although the cell may carry altered information, so long as the altered area remains unexpressed no change in phenotype will occur even when the cell divides in the normal course of events. Before a change in phenotype can be seen, a promoting event is required which will allow the altered area of genome to become inducible, *i.e.* the affected area must be morphologically altered into a transcribing rather than a non-transcribing form.

The action of promoters in carcinogenesis has been extensively studied in the skin, and we have extended some observations to the urinary bladder. In general, promoters increase the opportunity for abnormal phenotypic expression by accelerating the rate of mitosis; this also helps to propagate the tumour. In addition, by randomly altering gene expression, they increase the opportunity of expressing any altered information previously introduced into the genome by exposure to an initiator. Furthermore, by inhibiting terminal differentiation of the tissue, they increase the time required before a cell will pass into the "differentiate and die" pathway, and retain the cells for longer in the cell cycle where they can grow and divide. In the biogenesis of bladder cancer, vitamin A deficiency clearly acts as a promoting stimulus. Vitamin A deficiency causes an increase in DNA synthesis and increased mitotic activity in the basal cells of the urothelium which causes hyperplasia. Coincidentally with this rather nonspecific stimulation of cell division and tumour promotion, vitamin A deficiency also specifically affects the genes controlling the differentiation of the cell, so that bladder tumours in vitamin A-deficient animals are also heavily keratinized. The same is true where cancer growth is associated with mechanical irritation, as in the presence of a urinary calculus or in bilharzial bladder in which the wall is filled with calcified ova, for irritation predisposes to squamous metaplasia. In both these instances, keratinization is concomitant with but not an essential part of the biogenesis of the neoplasms. The keratinization demonstrates that vitamin A deficiency or irritation produce a disturbance in the normal control of transcription. If such factors also encourage random transcription of the genome, they will automatically increase the chance of transcribing potentially carcinogenic changes in the genome introduced by previous exposure to an initiator. We have demonstrated that saccharin also acts as a promoter of the growth of bladder cancer in animals previously subjected to an initiating dose of the carcinogen methyl nitrosourea (MNU) (Hicks *et al.*, 1975; Hicks & Chowaniec, 1977). These observations have now been confirmed by Friedell and his co-workers, using an initiating dose of another bladder carcinogen, the nitrofuran FANFT (Cohen *et al.*, 1978). It is not known how saccharin acts as a promoter, but undoubtedly tumours promoted by saccharin show a wide variety of differentiation patterns both within the tumour and in adjacent, non-neoplastic areas of the urothelium. This suggests that saccharin in some way disturbs the normal control of differentiation and, like vitamin A deficiency, encourages random gene transcription, thus increasing the chance of expression of any neoplastic modification of the growth-controlling genes introduced by pre-treatment with an initiator. Saccharin also causes urothelial hyperplasia in some but not all animals, and increased numbers of cells enter mitosis. Thus saccharin has two properties characteristic of skin promoters: it appears to increase the chance of random transcription of the genome and at the same time it encourages cell division, thus promoting and propagating tumour growth.

The final result of neoplastic transformation in any tissue is an altered phenotype, such that the cancer cell expresses certain properties which differ from those of normal cells from which they were derived. It appears to be a disease in which some concomitant phenotypic change in differentiation is obligatory. However, the changes which occur in a tissue during carcinogenesis may also illustrate many changes in differentiation which are not necessarily a part of the neoplastic growth syndrome. Promoters encourage random gene expression and the production of multiple phenotypes, among which may be the phenotype associated with cancer growth if the tissue has previously been exposed to an initiating carcinogen.

This work has been supported over a number of years by generous grants from the Cancer Research Campaign.

REFERENCES

Cohen, S. M., Arai, M., Jacobs, J. B. & Friedell, G. H. (1978) *Cancer Res.*, **39**, 1207.

Firth, J. A. & Hicks, R. M. (1970) *J. Anat.*, **107**, 192.

Hicks, R. M. (1968) *J. Ultrastruct. Res.*, **22**, 206.

Hicks, R. M. (1969) *J. Anat.*, **104**, 327.

Hicks, R. M. (1975) *Biol. Rev.*, **50**, 215.

Hicks, R. M. (1976) *Progress in Differentiation Research*. Amsterdam: North Holland Pub. Co. p. 339.

Hicks, R. M. & Chowaniec, J. (1977) *Cancer Res.*, **37**, 2943.

Hicks, R. M. & Chowaniec, J. (1978) *Int. Rev. Exp. Pathol.*, **18**, 199.

Hicks, R. M., Chowaniec, J. & Wakefield, J. St J. (1978) *Carcinogenesis, Vol.* **2**, *Mechanisms of Tumour Promotion and Co-carcinogenesis.* New York: Raven Press. p. 475.

Hicks, R. M., Ketterer, B. & Warren, R. C. (1974) *Phil. Trans. R. Soc. Lond. B.*, **268**, 23.

Hicks, R. M., Wakefield, J. St J. & Chowaniec, J. (1975) *Chem.-Biol. Interactions*, **11**, 225.

Severs, N. J. & Hicks, R. M. (1977) *J. Microscop.*, **111**, 125.

Yuspa, S. H., Del Sol, A. E., Morgan, D. L. & Bates, R. R. (1969) *Chem.-Biol. Interactions*, **1**, 223.